# Stereopsis: are we assessing it in enough depth?

**DOI:** 10.1111/cxo.12655

**Published:** 2018-01-27

**Authors:** Anna R O'Connor, Laurence P Tidbury

**Affiliations:** ^1^ Directorate of Orthoptics and Vision Science University of Liverpool Liverpool UK

**Keywords:** binocular vision, motion in depth, stereoacuity, stereopsis, strabismus

## Abstract

The assessment of stereoacuity is an integral part of the ophthalmic assessment, with the responses used to inform clinical management decisions. Stereoacuity impacts on many aspects of life, but there are discrepancies reported where people without measurable stereoacuity report appreciating 3‐D vision. This could be due, in part, to the presentation of the stimuli. A literature review was undertaken to evaluate current assessment techniques, how they relate to patient outcomes, identify the limitations of current tests and discuss how they could be improved. Recent evidence has been collated on currently available tests, used commonly within vision clinics, with normative data provided allowing responses to the tests to be interpreted. The relevance of the results is evaluated in relation to a range of outcomes, where a reduced level of stereopsis has a negative impact on the ability of an individual to perform many tasks, and can lead to an increase in difficulty interacting in the world. Current tests are limited in the aspects of stereoacuity they assess and their ability to precisely measure stereopsis. The world is not static, yet clinical tests are limited to measuring static stereoacuity, even though higher grades of depth perception can be identified in the presence of changing depth. Presentation methods of stereoacuity tests have remained similar over time, with a limited number of disparity levels assessed. New assessment methods are becoming available that include automated staircase testing to present multiple levels of disparity using digital technology. Current clinical tests are limited in their presentation, and are poor at detecting/measuring stereoacuity in those with limited stereopsis. Given the relevance of the stereoacuity measurement to management choices and functional outcomes, new testing methods would be beneficial to fully assess stereoacuity, both static and dynamic.

Only one eye is needed to perceive depth due to the multitude of monocular cues to the presence of depth, such as perspective, size, and order, as well as cues that include movement, such as motion parallax and looming.^1^ Therefore it could be argued that binocular depth perception is not important and does not need to be assessed. As reported by one ophthalmologist, ‘Stereoscopic vision is of little value except in a few occupations’, with the primary advantage of having two eyes being reported as having ‘a spare one’, in case of visual impairment.^2^ However, the complex two‐eyed visual system is designed not simply for redundancy, but primarily to allow both eyes to work together to extract information from the environment. Approximately 60 per cent of the human visual field overlaps, where greater peripheral vision is sacrificed in favour of information provided by binocular disparity, but this results in benefits to functional ability.

Stereoacuity is routinely measured clinically, and the tests currently used are accurate in identifying people with good levels of stereoacuity. However, a review of the literature highlights how clinical tests may incorrectly diagnose an individual as stereoblind.^1^ This is attributable to the tests’ lack of sensitivity to change and that binocular potential, in the presence of strabismus, is not fully assessed.

Given these issues, the aims of this review are to:

- evaluate the current clinical assessment of stereoacuity
  - with a focus on the assessment in children and the impact of the developing visual system
  - also, evaluating the discrepancy between patient reports and clinical measures
- discuss the relevance to patient outcomes
  - specifically in relation to patients with strabismus which is often accompanied by amblyopia
  - using infantile esotropia as an example for the impact of strabismus on stereopsis and evaluating the potential recovery
  - evaluating the impact of the presence of stereopsis on functional skills
- consider potential future developments.

Discussion throughout will include the relevance to clinical care.

## DEVELOPMENT OF STEREOPSIS

Given the potential for improving outcomes of deficits affecting the visual system is greatest during childhood, due to the plasticity, it is important to understand how binocular vision develops and know what a ‘normal’ response is across childhood. At birth, visual functions are poor but develop rapidly over the first few months of life, with appropriate stimulation.^3^ There are three critical periods identified in relation to visual development (development, sensitive and recovery), but the timing of each period varies in relation to each specific visual function.^4^

As disparity detection requires good acuity that is similar in each eye, stereopsis is not present at birth but develops following the improvement in visual acuity. Visual evoked potentials have demonstrated the development of stereopsis starts around three months of age, but with a rapid maturation in the first year of life.^5^ Over time there is further refinement, with normative data demonstrating an improvement over the first five years of life.^6^, ^7^ However, it is not possible to determine how much of this change is attributable to the improvements in cognitive ability as the assessment is typically undertaken using behavioural measures.

Neural plasticity is at its greatest during the sensitive period, where the developed function is susceptible and can be lost. This period can extend for many years, potentially into adulthood, but with an exponential decrease in plasticity associated with increasing age. The severity of the loss is greatest during the early development, which is clearly demonstrated in children with infantile esotropia.^8^ In this type of strabismus the onset is before six months of age, meaning the development of stereopsis is interrupted at a very early stage following a period of time when the eyes were aligned.

Re‐alignment of the visual axes can result in recovery of stereopsis, but is dependent on factors such as the timing of the strabismus onset and the duration of misalignment. Early‐onset strabismus has the lowest rates of recovery, as reported in children with infantile esotropia, in particular when the duration of misalignment is greater than three months.^9^ This makes the accurate assessment of stereopsis at a young age desirable, to inform a patient's management.

## MANAGEMENT OF LOSS OF STEREO

Infantile esotropia is a prime example of major deficit in binocular vision, where outcomes are judged in terms of alignment and the presence/level of stereopsis. The potential for the recovery of stereopsis is pivotal to the argument relating to the timing of correction in infantile esotropia. Reports do agree that the results are compelling showing treatment before the age of two years (and preferably younger) results in better levels of stereoacuity.^8^ Given that the rates of recovery of stereopsis are lower beyond a limited three month time frame between onset and correction,^10^ it is argued that it is beneficial to delay treatment until a later age, where there is less risk for a consecutive exotropia to develop. In addition any supplementary features, such as dissociated vertical deviation (DVD), typically start to develop around 18 months^11^ and can be treated in the same surgical procedure. However, evidence from animal models suggests that early correction resulting in stereopsis may be protective against the development of DVD.^12^ The key factor that tips the scales in favour of early surgery is the weighting attributed to having stereopsis, the potential benefits of which are discussed later.

Botulinum toxin (BT) has been advocated as a primary treatment for infantile esotropia,^13^ with stereoacuity reported in a long‐term follow up with stable alignment.^14^ While it could be postulated that the long‐term alignment may be attributable to a protective effect of having stereopsis, it cannot be differentiated from the impact of changes to the muscle function. The potential positive impact of BT has to be weighed against the risk of ptosis (27 per cent reported in a recent meta‐analysis^15^), which can result in amblyopia, and creating a barrier to binocular vision. So surgery continues to be the most common method of correction.

While an early‐onset constant strabismus can have a profound impact if not treated within a short window of time, intermittent deviations pose a much lower risk to losing stereopsis, where a delay in treatment may have little or no impact on the long‐term stereoacuity levels.^16^, ^17^ A reduction in near stereoacuity is often cited as a reason to intervene rather than observe in cases of intermittent exotropia; however, it may again be the duration of misalignment that is the crucial factor in the loss/recovery of stereopsis.^17^ This is supported by evidence demonstrating that the presence of stereopsis post‐operatively is associated with factors relating to the timing of the strabismus onset (later having a better outcome), the frequency of the deviation (intermittent being more likely to have a positive stereo response), strabismus duration less than 20 years, or unequal visual acuity.^18^

To inform the management choices and goal, stereoacuity is typically measured pre‐ and post‐operatively, with the results being used in combination with other clinical measures. It may be argued that the lack of any measurable stereopsis, even with the angle of deviation corrected, is a strong prognostic indicator of the post‐operative binocular status. As a result, the surgical aim is typically to leave the eyes in a slightly esotropic position, to account for the expected exotropic drift associated with age. While this strategy has the potential to result in less residual/consecutive exotropia, and resultant surgical procedures, the target angle which has the potential to result in stereopsis (and therefore maintaining long‐term alignment) may be smaller than the 10 dioptres frequently used to quantify a ‘good’ surgical outcome.^19^ Given the impact on the surgical choice, the information gained from the clinical assessment is very influential. However, if the tests used are not sufficiently sensitive to the detection of stereopsis, this approach could result in an unnecessary permanent deficit of binocular vision. Therefore, it is important to evaluate what the current clinical tests can and cannot tell us.

## CURRENT METHODS OF ASSESSMENT

There are many clinical tests on the market, all with the same basic principle of presenting a different image (half image) to each eye, but with a range of methods of presenting the disparity, for example polarising, anaglyph, lenticular or physical/‘real’ depth. In theory, the method of presentation should not influence the detection of disparity; however, normative data across tests vary, suggesting an impact of the presentation method.

A description that appears within the literature when describing tests of stereoacuity is the phrase ‘real depth’. This is generally used to describe the Frisby and FD2 tests, as the depth is physical. The term is also applicable to the original Howard–Dolman rod test and subsequent evolutions, where two or more rods can be displaced by incremental amounts until a difference in depth can be detected between them.^20^, ^21^, ^22^ These differ from other tests used in the clinic such as the TNO and Randot Preschool tests as they do not require a filter to separate the left and right half images to each eye.

The level of stereoacuity measured using tests with a filter of some description tends to appear ‘worse’, often attributed to the dissociative effect of the filters. However, it has been demonstrated that the dissociative effect is not an influencing factor in the different stereoacuity levels measured between ‘real’ and random dot tests, with the suggestion that a ‘real’ test measures a different aspect of stereoacuity.^23^, ^24^

Depth from disparity is determined by detecting the horizontal offset between the edges of stimuli, when the point of fixation is a single percept. The disparity in a ‘real’ depth plane is created by the horizontal separation between each eye creating two slightly different views of a scene. This feature makes the tests more susceptible to monocular cues, especially where a comparison can be made between two viewpoints observed at different times, that is motion parallax, exacerbated by the presence of an aperture such as the frame around the shapes in the FD2. Slight movement of either the test or observer can introduce this unwanted cue to the test.

Ignoring any monocular cues, which should be absent by design and protocol in all clinical tests of stereoacuity, all tests deliver two separate flat images containing no depth information to each eye. The combination of these images, and resolving of the correspondence problem, results in the perception of depth. This is in essence not different from any other method of presenting an image to each eye, other than the introduction of a filter to present the appropriate half image to each eye. The term ‘real depth’ is perhaps a misnomer, as depth is the percept, recognised through binocular disparity. If a description were required, we would suggest the use of the term ‘physical depth’ as it relates to the stimulus (object) rather than the percept of depth.

The Titmus circles/Wirt fly test is commonly used in vision labs and clinics around the world, especially as the fly element displays the largest level of disparity available in a commercial test (3,000″) and is volumetric (a virtual object) rather than a depth displaced plane. However, it is easy to guess the response due to monocular cues and especially due to familiarity with objects. The wings of the fly would appear in an expected location so indicating their location may not be an assurance of stereopsis. Two modifications have been suggested to improve accuracy, including rotating the book to a new orientation and repeating the assessment,^25^ or by using glasses with polarisers aligned the same way to provide a monocular‐only view for comparison,^26^ requiring the child to consistently provide a positive response only to the presentation with disparity.

Irrespective of the method of presentation, any assessment of visual function should fulfil the following criteria:

- have available normative data to facilitate interpretation of the responses
- low test–retest variability, to be able to detect changes in the clinical condition
- high sensitivity and specificity for the target condition(s)
- high testability (the number of people in a particular group that can successfully perform the test) for the target population.

In addition to these generic criteria, an additional factor for stereoacuity tests would be the lack of any monocular cues. The wide range of commercially available tests used within the clinical setting have varying levels of evidence to support their use, but often ease of use or personal preference is the deciding factor in a clinical setting. There is variability in the normative values obtained, both between and within tests. Some degree of the variability may be attributable to the threshold estimation method, but another important factor to note is the modification of the test, where the recent version of the TNO results in a lower stereoacuity response compared to the original test.^27^

**Figure 1:**
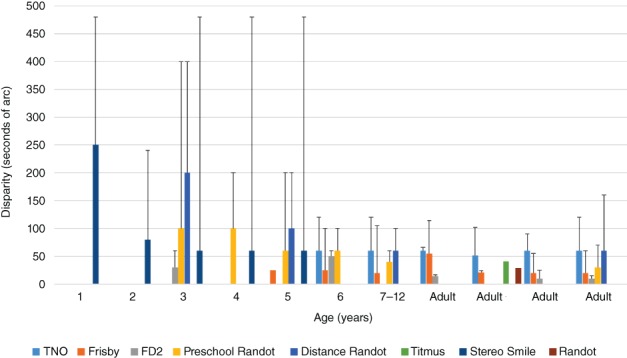
Normative data for a range of stereoacuity tests, grouped by age. Coloured bars indicate the mean value and the black bars indicate lower limit of normal values.^7^, ^22^, ^28^, ^29^, ^30^, ^31^, ^32^, ^33^, ^34^, ^35^

While the normative values, and lower limits (black bars in Figure 1), provide information regarding whether a single response is within normal limits, it is also important to be able to evaluate whether a change in stereoacuity at a subsequent visit is significant. This is typically based on the test–retest variability values, with values varying depending on the test, with identical scores on repeated testing being found in 25–73 per cent of children.^36^, ^37^, ^38^, ^39^ However, these studies agree that in older children there is little variation if they have no visual deficit.

The variability of stereoacuity in subjects with intermittent strabismus has been evaluated to determine a ‘normal’ level of variability in these cases,^40^ reporting a higher level of change being needed to detect a significant change. However, this is based on the supposition that strabismus with varying stereoacuity is a ‘normal’ state and does not require intervention. Given that people with good levels of stereoacuity show little variation on repeated testing, it suggests that stereoacuity should not vary. Therefore, any variation could be considered ‘abnormal’ and detrimental to the person.^41^, ^42^

In a clinical setting prior to strabismus surgery the aim may not be to measure what stereoacuity the patient has, but what potential they have following surgery. The investigation of binocular potential in strabismic patients is limited and may only involve presenting a target at the corrected angle to assess if any diplopia is elicited. However, in the presence of amblyopia, changing the position of the eye may not provide the appropriate stimulus to use both eyes together, as the fixating eye dominates. The use of neutral density filters could offer a balancing similar to that used in the binocular treatment of amblyopia, but the prisms used to correct the angle can cause optical distortions which may limit the effect. To then assess binocularity using a stereoacuity test that requires glasses could prove challenging.

The synoptophore offers a solution to this, as the angle of deviation can be easily corrected by repositioning the tubes, the balance between the stimuli strength can be adjusted using the rheostats to reduce the luminance of the fixing eye tube, and the stereopsis slides can provide an indication of binocular depth detection. The stereopsis slides are typically limited to an indication of depth order or direction, although Braddick Random Dot graded stereo slides are available, offering a testable range of 720″ to 90″ over six pairs of slides. It is also possible to modify the synoptophore to use digital screens to display the desired stimuli, including targets with numerous levels of disparity to assess stereoacuity.

Achieving high levels of testability in young children is challenging for any measure of visual function, as they require the child to understand, interpret and communicate their perception. Testability rates in children under five years of age vary; however, rates over 90 per cent testability are reported as young as age six months using the Lang II test, based on eye movement responses.^43^ Other tests, requiring a verbal or pointing response, do not reach this high level of testability (over 90 per cent) until around four to five years of age, with low levels of testability in children aged three to four years (31–81 per cent for Random Dot test).^43^, ^44^, ^45^ While the Lang II has high testability at a young age, there is a trade off with accuracy, with a sensitivity for binocular dysfunctions as low as 21 per cent.^46^

While high sensitivity and specificity are another desirable feature of clinical tests, data show there are varying levels,^47^, ^48^ in part due to the condition(s) that the tests are being evaluated to detect. Typically strabismus and/or amblyopia are used as the target conditions, as there is a correlation between these conditions and reduced stereoacuity. There is a known correlation between amblyopia severity and stereoacuity levels; however, this is not a perfect correlation.^49^ The same applies to strabismus, which may be intermittent and have normal stereoacuity at one distance. Consequently, using these target conditions will mean the sensitivity/specificity data is not demonstrating the test's efficacy to detect reduced stereoacuity. Overall, all clinical tests have limitations, particularly in the younger age group, but in older children they can be very accurate. The important point to remember when interpreting a response is that it is specific to that test and the age of the patient.

## IMPACT OF AMBLYOPIA

While analysis of the new binocular amblyopia treatments is beyond the scope of this review, it is important to consider their relevance to the improvement or restoration of stereopsis (reviewed in Foss 2017^50^). Occlusion has been the primary treatment for amblyopia for decades, but presents challenges in respect to compliance, and while occlusion does improve visual acuity, it may be at the expense of binocular vision. In contrast, dichoptic binocular amblyopia therapy promotes the use of the amblyopic eye by diminishing the signal to the fixing eye, by reducing contrast or luminance,^51^, ^52^ or presenting different parts of the task to individual eyes.^53^

The shuttering of active 3‐D displays is comparable, halving the time the signal is presented to the fixing eye. Even in the case of strabismic viewers, who are unable to take up fixation, binocular information may still be extracted through recognition of motion in depth. In a range of small‐scale studies into the binocular treatment of adult amblyopia, improvements in stereoacuity between 26″ and 1,667″ were found.^51^, ^54^, ^55^ These levels were still achieved by participants at the end of their treatment regimens when the binocular balancing was removed, with some subjects showing an improvement in stereoacuity level, with little improvement in visual acuity.

## WHY DO PEOPLE WITHOUT MEASURABLE STEREO REPORT SEEING IN 3‐D?

Many patients have no measurable stereoacuity on clinical tests but there are anecdotal reports of 3‐D depth being perceived by these people. This questions whether the tests are accurate or if the subject is perceiving some cue to depth not present in the clinical tests.^56^, ^57^, ^58^, ^59^ The assertion that vivid 3‐D vision can be experienced with just one eye is arguably a matter of personal opinion, perception and state of binocularity.^56^, ^60^

The discrepancy between clinical tests and the subject/patient response could be attributed to current clinical testing methods only assessing one aspect of depth perception. Technology such as 3‐D televisions and handheld games are very different from this, not only in what is shown, but in the presentation method. While the glasses (Real‐D system) used most commonly at the cinema are passive polarising (similar to the Randot, but circular rather than linear polarising), there is still an active element. The polarisation difference is created by a liquid‐crystal display filter placed in front of the projection lens, which determines which frame is shown to each eye by alternating polarity. These changes in viewing eye per frame may be imperceptible; however, each eye is not being presented an image at the same point in time, as is true of most clinical tests.

The Baylor Visual Acuity Tester is a rarely used clinical test utilising active shutter glasses, which is similar to the technology used for ‘active’ home 3‐D televisions;^61^ however, this test differs in that the glasses have a very low refresh rate per eye of 30 Hz, whereas modern active 3‐D televisions have a minimum of 60 Hz refresh rate per eye. Autostereoscopic screens, such as that of the Nintendo 3DS for which glasses are not required, are similar to the Lang stereotest, and passive 3‐D television screens, in which a filter on the screen determines the polarisation of each vertical line on the screen, allowing an image to be presented to both eyes at the same time.

Given the significant differences in presentation method and content, variability between clinical measures and 3‐D entertainment media is expected; however, the magnitude of that variation is surprising. To explore this discrepancy, we performed a series of tests to attempt to quantify this effect, which showed that in the presence of any measureable stereoacuity, subjects reported the impression of convincing depth when viewing the 3‐D videos.^62^ In a subgroup of seven non‐binocular subjects, no subject provided a clinically measurable level of disparity; however, responses to 3‐D entertainment media tasks ranged from nil to ‘appears very 3‐D’, and the depth order of five objects within a number of set scenes were correctly identified up to 55 per cent of the time (chance of getting it correct was > 20 per cent). This supports the notion that a negative response on a current clinical test does not necessarily reflect an absence of stereopsis. We rarely encounter purely static presentations of depth information in the world, yet it is the only element of stereopsis we assess.

## MOTION IN DEPTH

**Figure 2:**
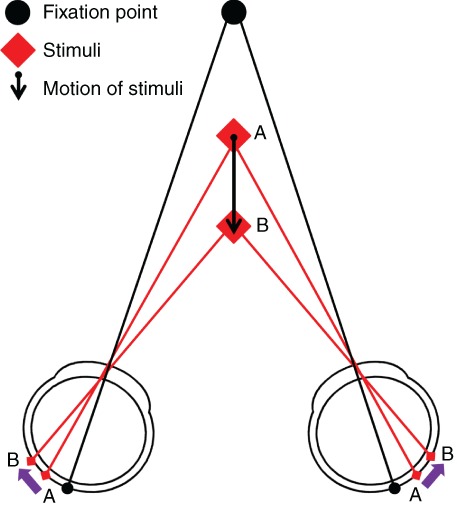
Diagram of disparity change. As the object ‘A’ moves toward the eyes to position ‘B’, its binocular disparity increases as its position on the retina changes. The purple arrows show direction of motion of the real object and the projection of the object on the retina.

While clinical assessment of stereoacuity is limited to a fixed static presentation, binocular depth information is typically encountered in a dynamic form, both in 3‐D entertainment and real life. Indeed stereopsis is most likely to be utilised within everyday activities when motion is involved. This facet of binocularity is known as motion in depth. There are two mechanisms that result in the perception of binocular motion in depth: changing disparity over time (CDOT) and interocular velocity differences (IOVD). The mechanism that detects CDOT relies on the interpretation of changes in the separation between any spatially corresponding points in the right and left eye (Figure 2). A CDOT stimulus is perceived as movement through depth (z‐motion, that is motion toward or away from the observer) through the recalculation of disparity and recognition of a change in disparity over time, providing information on changing depth.

**Figure 3:**
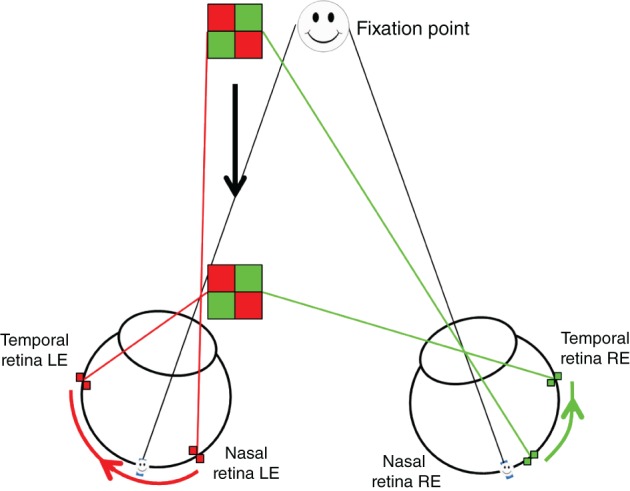
Diagrammatic representation of the interocular velocity differences (IOVD) cue. The non‐spatially corresponding points undergo differing motions across each retina. The red elements of the stimulus have further to travel and therefore move at a greater speed – the velocity is greater. In contrast the velocity of the green elements is smaller, moving in the opposite direction, at a slower speed. The difference in velocity between the motions across the retina is interpreted as motion through depth: the object is perceived to approach the observer.

The second mechanism extracts the IOVD between the two eyes. The IOVD mechanism does not require spatially matching points between the two retinas; rather it utilises motion of individual points across each retina separately, and the difference in velocity between the two eyes is used to infer depth (Figure 3). For example, an object which moves straight toward an observer will result in rightward retinal motion in the right eye and in leftward motion in the left eye. Comparing these two velocities is informative about the change in depth of the object.

While there is evidence for two distinct mechanisms processing these cues (CDOT and IOVD),^63^, ^64^, ^65^, ^66^, ^67^, ^68^ under natural viewing conditions these two cues are unlikely to occur in isolation and performance is better when both cues are present. The more robust cue for the extraction of dynamic depth tends to be CDOT, with only small subsets of individuals able to use the IOVD cue in isolation.^1^, ^64^, ^66^, ^69^, ^70^

## THE DETECTION OF DEPTH WITH CHANGING DEPTH

There are few studies that have directly considered the detection of depth in moving stimuli, with the majority of studies considering the perception of direction of motion in depth. Individuals tend to perceive a greater amount of depth in stimuli that move through depth. When asked to match the amount of depth apparent in an approaching stimulus (communicated by changes in disparity/IOVD only) participants consistently matched lower amounts of disparity in the dynamic stimuli, to stimuli with static disparity.^70^ Earlier reports have not conclusively demonstrated any advantage to movement through depth.^71^, ^72^

Studies within our own lab have investigated the contribution of motion in depth and other dynamic cues to the detection of depth, using well‐controlled stimuli that are directly comparable.^1^, ^69^ The same stimuli design and testing methodology were used for all conditions, demonstrating that depth is more apparent when an object appears to approach an observer. Thresholds for depth detection were superior in the stimuli that included a change in z‐location (amount of depth), more so than the static presentation. This shows that the visual system is able to utilise binocular information to identify depth in moving targets, with less disparity than is required to identify static depth.

## STEREOACUITY AND MOTION IN DEPTH

Stereoacuity is traditionally considered as the threshold measure of how well an individual can interpret binocular disparity as perceived depth, by determining the spatial correlation of points projected onto the retina. As a physical object moves toward or away from an individual, a number of factors change, including monocular cues, and the two binocular cues to depth. Any point forward of where the eyes are fixated provides crossed disparity, that is, these points are projected on the temporal retina of both eyes. This is binocular disparity, as the corresponding point to the temporal retina of one eye, is the nasal point of the other eye.

## THE DETECTION OF MOTION VERSUS THE DETECTION OF DEPTH

The ability to determine the motion of a target as it moves through depth has been demonstrated in the absence of measurable static stereopsis using standard clinical tests.^73^, ^74^, ^75^, ^76^ All of these studies demonstrate the potential of subjects with no measurable static stereoacuity to provide a response based on binocular processing when the stimuli contain stereomotion. However, there are a number of barriers in previous investigations that do not allow us to be confident that depth detection from stereomotion is superior to static depth detection. The range of disparities presented differ between the static and dynamic stimuli, use very different display methods and types of stimuli. The enhanced perception of depth reported by clinically diagnosed stereoblind subjects, could also be attributed to peripheral cues; findings in the central visual field show that while 40 per cent of subjects could detect static depth, only 24 per cent were able to detect depth from stereomotion.^77^

## IS IT IMPORTANT TO IDENTIFY STEREOPSIS (OR THE POTENTIAL FOR STEREOPSIS)?

Stereopsis is an ability not limited to depth detection, but contributes to camouflage breaking, as demonstrated by the Julesz stereogram^78^ where form can be detected in noise, based on binocular disparity, which is exploited in many of the clinical tests of stereopsis used in the clinic today. Many members of the animal kingdom have stereopsis, typically with the aim of detecting predators or prey which are camouflaged within their environment.^79^ Moving away from animals, humans have also made use of the ability to break camouflage. The use of sequential aerial photography during the First World War, demonstrated how a stereoscopic view of terrain could highlight features previously undetected during monocular viewing, revealing variations in terrain and edges of camouflaged buildings.^80^ The ability to perceive the depth edge and/or volume of an object, has clear benefits when interacting with objects and navigating in 3‐D space.

Research to understand the neurological basis of stereopsis dates back over a century, but the relevance of stereopsis to everyday living was largely ignored in early vision research or considered irrelevant. Anecdotal evidence documented the impact on individuals;^81^ however, since the 1990s there has been a wealth of research evaluating the functional relevance of stereopsis. Traditionally strabismus surgery was termed as a ‘cosmetic’ procedure, but evidence clearly demonstrates that correcting the deviation has a significant impact on a patient in many aspects of life, in particular the psychosocial domains. Given the relevance to the availability of funding for strabismus surgery, it is of significant interest to determine the impact on all aspects of life. The effects of decisions regarding management in childhood are far reaching, impacting on career opportunities, and it is important to consider how the presence of stereopsis impacts on daily life and whether it can be recovered.

‘Stereo Sue’ sparked an interesting debate into whether stereopsis is recoverable in adults who have previously never demonstrated any measureable stereoacuity.^82^ This challenges the notion that stereopsis has a fixed recovery period limited to childhood. The recovery of stereopsis following treatment in adults may be possible, but exercises such as those described by Susan Barry, are not typically attempted in adults with long‐standing early‐onset strabismus due to the perceived risk of intractable diplopia. However, based on recent prevalence data of intractable diplopia,^83^ this may be an overstated risk.

Since the utilisation of disparity information is greater at near, it is anticipated that fine motor skill tasks, such as bead threading and ball catching, would be affected by a lack of stereopsis. This is supported by a wealth of evidence where a significant impact in speed and accuracy occurs under monocular conditions,^3^, ^84^ but there is also a reduction when stereoacuity is below normal levels. Analysis of the kinematics of the hand movements demonstrate that in reaching and grasping tasks, the hand aperture is wider and inaccurate, taking longer overall to reach the target but in particular slowing on the final approach. However, the relationship between stereoacuity and motor skills is not linear, with the absence of stereoacuity having a much greater impact, suggesting that the presence of some stereopsis is better than none.^85^

Although utilisation of disparity information is greatest at close range, the impact on functional ability is not limited to near tasks. Those with reduced/absent stereoacuity also have measurable differences in gait, demonstrating a more cautious approach with higher toe clearance and increased hesitation around steps.^86^ This may not present a problem to someone without walking difficulties, but as stereoacuity reduces in later life, and it is reported that abnormal stereoacuity is a risk factor for falls in the elderly, the impact of poor stereoacuity significantly escalates.^87^, ^88^ Additionally, as sensitivity to contrast decreases with age, abnormal stereoacuity can make it even more difficult to identify the difference between a kerb‐edge and the road. As with random dot stereograms, where the edges of a shape cannot be perceived without recognising disparity to break camouflage, an inability to extract depth information will prevent identification of trip or fall hazards.

Surgical alignment may result in improved motor skills, although it is challenging to evaluate this in children given the wide range in ages at which developmental milestones are reached. In a population of children with infantile esotropia, where sensorimotor and gross motor development was delayed prior to surgery, participants showed an improvement post‐operatively with their skills being no longer delayed compared to children without strabismus.^89^ This is encouraging; however, it cannot simply be attributed to an improvement in binocularity, as it is not known what the developmental trajectory of these children is and whether they simply have a later developmental pattern irrespective of surgery.

One population where there is a significant increase in the rates of stereo impairment is in children with learning difficulties.^90^ Given their associated challenges with motor skills, the impact of the loss of stereoacuity could be having a bigger impact, where a child without motor function problems could compensate for the loss of binocular vision. It could be argued that improving stereopsis may aid in development, but determining the impact of any intervention would be problematic.

While 3‐D in the home has not become as prevalent as hoped by technology manufacturers, content is produced for the cinema where 3‐D films are still popular. Similarly, 3‐D has found a resurgence in the form of virtual reality, ranging from the cheap Google cardboard viewers to the full‐room scale tracking HTC Vive. These immersive technologies and experiences at the cinema, can be enjoyed through monocular cues to an individual who has no binocular vision; however, the compelling effect enjoyed by their peers may be lacking, potentially along with performance in games that require depth judgements. As well as social uses for these technologies, educational use is increasing at all levels of education. Rather than watching a video, children could put on a headset and be part of a scene providing an immersive experience. Within our university orthoptics course, students are able to view and interact with an eye the size of a room, holding and pulling muscles to see how the eye position is affected.

Current research has focused on what is easily quantifiable, typically how fast or accurate a person is at a given task, and the numerous reports agree that high‐grade stereoacuity is beneficial.^84^, ^85^, ^91^, ^92^, ^93^ However, the next step required in research is to evaluate whether the patients perceive this reduction and if it has impacted on their quality of life. It is clear that people who had good stereoacuity but lost it due to pathology in one eye, do perceive the impact, but can adapt, to a degree, over time. However, with a childhood strabismus prevalence of 4–7.5 per cent,^94^, ^95^ there are potentially millions of adults who have never had stereoacuity.

The question remains, how does the absence of stereoacuity impact on their daily lives? Extrapolating from the data on functional skills, it would be anticipated that there would be some influence; however, many jobs do not require high levels of fine motor skills. Also, sporting preferences may be self‐selecting: if you are poor at ball sports you may choose to avoid them. Evidence regarding whether the restrictions on employment or hobbies are perceived by the person, and whether they impact on their quality of life, or to what extent, is lacking. There are data to show the impact of strabismus on the quality of life, resulting in poor or absent stereoacuity, but it is challenging to isolate the impact from the lack of binocular vision from the impact of the appearance.

## FUTURE METHODS OF ASSESSMENT

While the printed format of tests of stereoacuity offer the ability to assess relatively good levels of stereoacuity, they can be insensitive to changes in stereoacuity due to the large differences in disparity levels presented, where cost and weight implications are prohibitive for printing a higher number of pages. Or, in the case of the Frisby test, a requirement would be to test at an infinite range of distances to accurately measure threshold.

The ceiling levels tested are also limited in some tests, given that up to 63 per cent of strabismic patients were able to identify disparity of 2,500″ while only five per cent could identify disparity of 800″ (the ceiling of a number of tests),^96^ it is clear that current tests are limited in their ability to detect the presence of any level of stereoacuity. The precision of the clinical tests are limited by the large variation of disparity between levels. In contrast, the rod‐based tests are only limited by the increments of separation, allowing a vast range of disparity to be presented. While this may be more accurate, as with presenting the Frisby plates at additional distances, the time and calculation required to test and score multiple levels restricts use in the clinic.

The use of digital displays allows the display of multiple levels of disparities, limited only by screen resolution at the fine end and screen width at the coarse end. As these screens are controlled by a computer, the implementation of staircase methodology can be used to automatically control the multiple levels of disparity presented using passive and active display technology,^61^, ^69^, ^83^, ^97^ allowing the calculation of a true threshold measurement. Currently there are packages offering multiple tests of visual function, such as the ‘Thomson Software’, but these do not fully exploit the capabilities of a computer, rather they simply display a digital version of the printed test.

The Asteroid test (Accurate STEReotest On a mobIle Device), currently in development, has been designed to address some of the issues identified in current clinical tests. The stimuli are presented on an autostereoscopic 3‐D tablet, using the device's camera to actively monitor test distance and adjust the disparity accordingly, using anti‐aliasing to present subpixel levels of disparity.^98^ In addition, an adaptive staircase is utilised for threshold calculation and the whole test is presented in a game format designed to be more engaging. If computerised tests such as the Asteroid were to include a more complete package of visual function assessment implementing staircase protocols, our ability to accurately and precisely assess all visual functions would increase.

## METHODS OF GENERATING 3‐D IMAGES

There are a large number of screens commercially available that are used to research 3‐D vision, and that could be clinically. However, there are a number of limitations to these screens, including resolution, sensitivity to viewing position, cross talk and hardware capabilities.

**Figure 4:**
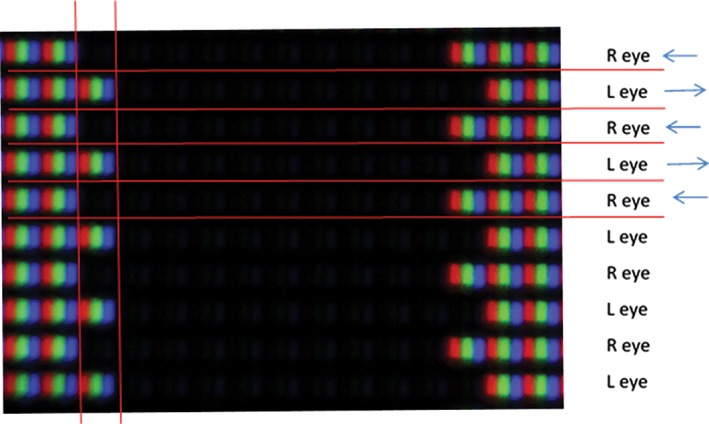
Macro photograph of a 10 by 10 pixel black square with one pixel of on‐screen disparity. Horizontal lines separate left and right eyes, vertical lines show one pixel. Additional: each red (R), green (G) and blue (B) oblong is a sub‐pixel of one of the pixels described by (for example) a television manufacturer. A ‘full HD 1,080p’ television has 1,080 of these pixels rows vertically, and 1,920 horizontally (made up of subpixels 1920R, 1920G, 1920B). In the figure, as the RGB subpixels are the same intensity, that pixel will appear white. The one pixel disparity between the left and right eye lines will result in the perception of depth, by capable individuals.

An example of a commonly used passive 3‐D screen is shown in Figure 4. Each alternate line on the screen can only be seen by the corresponding eye when wearing the corresponding 3‐D glasses. Each red, green and blue triplet is one pixel. The black line between each row of pixels is designed to prevent ‘cross talk’, that is, transmission of the signal meant for the right eye to the left eye (and vice versa). However, this is dependent on the observer being well aligned with the screen, as not only is this vital to reduce any cross talk, but also to ensure that the correct eye receives the correct half image, so that the stimuli ‘pops‐out’ of the screen with crossed disparity rather than into it (uncrossed disparity).

Figure 4 produces an amount of crossed disparity, by artificially adjusting where the objects image falls on the retina. The fixation point must be the screen plane (backed up by a fixation target), otherwise the image will be slightly blurred. Therefore the image of the black square falls temporal to the fixation point on each retina.

The design of a typical wide screen desktop PC monitor has a resolution of 1,920 pixels wide by 1,080 high and has a visible screen width of 0.51 m. When viewed at 3 m, a shift of one pixel gives a disparity of 0.005**°** or 18.09″ where one arc second is 1/3,600th of a degree. Given that adult thresholds are below 18″, this results in a floor effect. However, anti‐aliasing (where shifts in the luminance of the pixels at the edge of the stimuli are augmented) can create smaller disparity shifts than whole pixels allow.^98^ The other way to improve the sensitivity and range of an electronic display‐based stereoacuity test is to increase the number of pixels while reducing their size. While the displays themselves exist to provide these features, the hardware required to control such a large number of pixels is limited by availability and high cost.

## CONCLUSION

Current clinical assessments of stereoacuity are effective at detecting good levels of stereoacuity, with data available to evaluate whether the response is normal, or represents a change in the clinical condition. However, they do not accurately reflect a person's perception of stereopsis in real life, in particular due to the small, flat, static nature of the stimuli. Given that the evidence regarding the importance of stereopsis to motor skills, stability of eye alignment, and quality of life, development of tests to evaluate all aspects of stereopsis would be extremely beneficial. In addition, this evidence also strengthens the argument to target accurate alignment at a young age, giving the child the best opportunity to develop stereopsis and all the resultant benefits.
